# Emotional Faces in Symbolic Relations: A Happiness Superiority Effect Involving the Equivalence Paradigm

**DOI:** 10.3389/fpsyg.2019.00954

**Published:** 2019-04-30

**Authors:** Renato Bortoloti, Rodrigo Vianna de Almeida, João Henrique de Almeida, Julio C. de Rose

**Affiliations:** ^1^Department of Psychology, Universidade Federal de Minas Gerais, Belo Horizonte, Brazil; ^2^Instituto Nacional de Ciência e Tecnologia Sobre Comportamento, Cognição e Ensino (INCT-ECCE), São Carlos, Brazil; ^3^Department of Psychology, Universidade Federal de São Carlos, São Carlos, Brazil

**Keywords:** symbolic behavior, stimulus equivalence, implicit relational assessment procedure, facial expressions, happiness superiority effect

## Abstract

The stimulus equivalence paradigm presented operational criteria to identify symbolic functions in observable behaviors. When humans match dissimilar stimuli (e.g., words to pictures), equivalence relations between those stimuli are likely to be demonstrated through behavioral tests derived from the logical properties of reflexivity, symmetry, and transitivity. If these properties are confirmed, one can say that those stimuli are members of an equivalence class in which each member is substitutable for the others. A number of studies, which have established equivalence classes comprised of arbitrary stimuli and pictures of faces expressing emotions, have found that valences of the faces affect the relatedness of equivalent stimuli. Importantly, several studies reported stronger relational strength in equivalence classes containing happy faces than in equivalence classes containing angry faces. The processes that may account for this higher degree of *relatability* of happy faces are not yet known. The current study investigated the dynamics of the symbolic relational responding involving facial expressions of different emotions by means of the Implicit Relational Assessment Procedure (IRAP). Participants were 186 undergraduate students who were taught to establish two equivalence classes, each comprising pictures of faces expressing either happiness (for one class) or a negative emotion (for another class), and meaningless words. The IRAP effect was taken as an index for the relational strength established between equivalent stimuli in the different equivalence classes. The dynamics of arbitrary relational responding in the course of the four IRAP trial types revealed that the participants exhibited a stronger IRAP effect in trials involving the happy faces and a weaker IRAP effect in trials involving the negative faces. These findings indicate that the happy faces had higher impact on the symbolic relational responding than the negative faces. The potential role played by the orienting function of happy vs. negative faces is discussed. By considering other studies that also reported a happiness superiority effect in other contexts, we present converging evidence for the prioritization of positive affect in emotional, categorical, and symbolic processing.

## Introduction

Humans seem to adapt to the environment in ways that are intrinsically symbolic, flexible, and generative (Barnes-Holmes et al., 2016). Despite the apparent agreement on this human feature, difficulties arise when one tries to distinguish between truly symbolic behaviors and non-symbolic interactions (Deacon, 1997). Sidman (1971) was pioneer in developing a behavioral analysis of derived relations (cf. Critchfield et al., 2018; see also Sidman and Tailby, 1982; Sidman, 1994). The Sidmanian paradigm of stimulus equivalence proposed that derived equivalence relations provide a basic functional account for the establishment of symbolic meaning.

Sidman and Tailby (1982) used the mathematical definition of an equivalence relation to provide operational criteria determining whether a relation between stimuli established in the lab is a relation of equivalence. For instance, a relation r between stimuli A and B (ArB), and between B and C (BrC), may be established by different procedures, such as matching-to-sample (e.g., Sidman, 1971), stimulus pairing (Leader et al., 1996), or a go/no-go procedure (Debert et al., 2007). If the relation r is an equivalence relation, training ArB and BrC should generate derived relations indicative of transitivity (ArC), symmetry (BrA and CrB), and reflexivity (ArA, BrB, and CrC), as well as combined symmetry and transitivity (CrA). These behavioral indicators of the logical properties of reflexivity, symmetry, and transitivity are used to infer that A, B, and C constitute a class of equivalent stimuli. Sidman (1994) stated that the mutual substitutability implied in the equivalence paradigm specifies “one way that symbols do become established as such, one way that words can come to “mean” what they “stand for” (p. 563).

Subsequent theoretical and empirical work has shown that other relational aspects need to be considered for a more complete account of derived relational responding (e.g., Hayes et al., 2001). Nevertheless, research on stimulus equivalence has enabled researchers to create artificial symbols in the laboratory. These artificial symbols can substitute for their referents, acquiring their psychological functions in a process that has been called symbolic generalization (e.g., Dymond et al., 2015; Bennett et al., 2015), or transfer of stimulus functions (e.g., de Rose et al., 1988; Gatch and Osborne, 1989; Barnes and Keenan, 1993; Dougher et al., 1994; Dymond and Barnes, 1994; Perez et al., 2017). The “symbolic status” of these artificial symbols has been demonstrated by several methods, such as lexical decision tasks (Barnes-Holmes et al., 2005; Bortoloti and de Rose, 2011a), semantic differential ratings (Bortoloti and de Rose, 2009), the Implicit Association Test (O’Toole et al., 2007), the Implicit Relational Assessment Procedure (IRAP) (Bortoloti and de Rose, 2012), semantic false memories (Guinther and Dougher, 2014), and Event-Related Potentials (Barnes-Holmes et al., 2005; Haimson et al., 2009; Bortoloti et al., 2014; Tabullo et al., 2015).

Different studies have shown that the relational strength (or relatedness) of members of equivalence classes vary as a function of several experimental parameters, such as the nodal distance between them (Fields et al., 1995; Bortoloti and de Rose, 2009), the simultaneous or delayed MTS employed in relational training (Bortoloti and de Rose, 2009, 2011b, 2012), and the amount of baseline training (Bortoloti et al., 2013), as well as the time elapsing between training and testing sessions (Silveira et al., 2016). Therefore, the paradoxical fact that stimuli regarded as equivalent may differ in relational strength has been pointed out by some investigators (e.g., Bortoloti and de Rose, 2011b; Doran and Fields, 2012).

Recent research has shown that the inclusion of a pre-experimental meaningful stimulus in an equivalence training influences likelihood of class formation (Fields et al., 2012; Fields and Arntzen, 2018) and also influences the relational strength within the class (Bortoloti and de Rose, 2012). A number of studies that established equivalence classes comprised of arbitrary stimuli and pictures of faces expressing emotions have found that valences of the faces affect the relatedness of equivalent stimuli. Bortoloti and colleagues (e.g., Bortoloti and de Rose, 2009, 2011b, 2012; Bortoloti et al., 2013) reported stronger relational strength in equivalence classes containing happy faces than in equivalence classes containing angry faces; Silveira et al. (2016) reported stronger stability in equivalence classes containing happy faces. None of these experiments was originally designed to compare the relational strength determined by facial expressions with different valences, but all of them showed what could be described as a *happiness superiority effect*. Processes that may account for this higher degree of *relatability* of happy faces are not yet known.

The current study sought to investigate the consistence of the happiness superiority effect with a larger sample of participants, and also tried to account for the impact of different types of facial stimuli on the symbolic relational responding induced in laboratory. The experimental design involved the application of the IRAP (Barnes-Holmes et al., 2010) to analyze the dynamics of the relational responding involving emotional faces and pseudo-words after equivalence training had simulated symbolic relations between these stimuli.

The IRAP has typically been used to measure brief and immediate relational responding that the participants have learned throughout their history of social interactions. It is assumed that the faster the response, the stronger is the participant’s attitude toward the relation presented on the screen. As hypothesized for other *implicit measures* (Cummins et al., 2018), the IRAP allows researchers to determine the existence and strength of relations between stimuli.

The IRAP involves the simultaneous presentation of a *label*, a *target*, and two *relational terms*. The experimenter can work with various labels and targets that alternate along successive trials. The participant is required to respond by pressing a key that relates label and target in a predefined way along blocks of trials that sometimes cohere and sometimes do not cohere with the presumed learning history of the participant. In general, experimenters arbitrarily set the relations in the *consistent condition* blocks of trials as the ones to which participants are expected to respond faster than to the ones in the *inconsistent condition* blocks of trials, depending on coherence with presumed history of social interactions. The difference between response latencies in the consistent and inconsistent tasks is called *IRAP effect*. Specifically, a difference score, based on the response latencies divided by the pooled standard deviation of response times across the consistent and inconsistent blocks, is used to infer the biases regarding the relation specified on the screen.

The IRAP has been used largely as a type of psychometric instrument for the measurement of implicit cognition (e.g., Hughes and Barnes-Holmes, 2011; Carpenter et al., 2012; Rabelo et al., 2014). More recently, several studies have demonstrated that the IRAP is also useful for exploring and analyzing the dynamics of arbitrary relational responding (e.g., Oliver, 2014; Finn et al., 2016; Maloney and Barnes-Holmes, 2016; Finn et al., 2018). Oliver (2014), for instance, asserted that features such as *coherence* to the history of reinforcement, *complexity* of the stimulus relationship, and the level of the participant’s experience with the stimulus relationship (*derivation*) presented on the screen might all influence response latency in IRAP trials. Consistent with this view, Finn et al. (2018) proposed that interactions between the function of the stimuli, the relationship between them, and the response options presented on the screen might account for different patterns of IRAP performances. In this sense, if an IRAP trial component has, for instance, a stronger orienting function, this feature will influence the dynamics of the arbitrarily applicable relational responding in the course of the IRAP trials. By considering the functions and interactions between the IRAP elements proposed by Finn et al. (2018), it would be possible to capture the strength of a given pattern of relational responding *in flight* (cf. Barnes-Holmes et al., 2016).

The aim of the present study was to capture the relative strength of experimentally induced relational responding by means of the IRAP. Participants were 186 undergraduate students who were submitted to two experimental phases. The first phase established two classes of equivalent stimuli involving pseudo-words and pictures of human faces expressing emotions. One of the classes was comprised of pictures of faces expressing happiness and the other was comprised of faces expressing a negative emotion, which was sadness for Subgroup 1, fear for Subgroup 2, disgust for Subgroup 3, and anger for Subgroup 4. In Phase 2, pseudo-words and faces were, respectively presented as *label* and *target* in IRAP tasks. The IRAP effect was taken as an index for the relational strength established between equivalent stimuli in the different equivalence classes. We had predicted that, if positive and negative emotional faces differently influenced the strength of experimentally simulated symbolic relations, this differential effect could be indexed by means of the IRAP effect. Part of the participants from each subgroup were submitted to the IRAP immediately after the relational training, and part were submitted to the IRAP one week later, in order to investigate the stability of the equivalence relations over time.

## Materials and Methods

### Participants

Participants were 186 undergraduates (59 males), students in a Brazilian university. Their native language was Portuguese, and they were not familiar with stimulus equivalence, IRAP, or related phenomena, concepts, and procedures.

All procedures performed in this study were in accordance with the ethical standards of the Brazilian National Health Council. The protocol was approved by the Federal University of Minas Gerais ethical committee. Participants were informed through a disclosure statement, provided to them at the beginning of the study, that they would serve as participants in an experimental simulation of symbolic relations. Participants were informed that their completion of the study was part of their training as experimental researchers since they would use similar procedures later in the semester in their own experiments, but they could quit their participation at any time. Information on all known risks and benefits of the study as well as confidentiality procedures was provided.

### Equipment, Setting, and Stimuli

Sessions were conducted collectively, with 10–20 participants, in a 7-m × 12-m laboratory facility equipped with 32 standard desktop computers. Each participant worked alone on a single computer. These computers were equipped with software for the matching-to-sample procedure and also with the IRAP software.^1^ Each matching-to-sample trial displayed five white windows (6 cm × 6 cm) on a gray screen, one at the center and one near each of the screen’s corners; participants responded by moving the computer’s mouse to position a cursor on a window and then clicking the mouse’s button. Each IRAP trial displayed two stimuli and two response options on a white screen; participants responded by pressing two keys on the computer’s keyboard.

**FIGURE 1:**
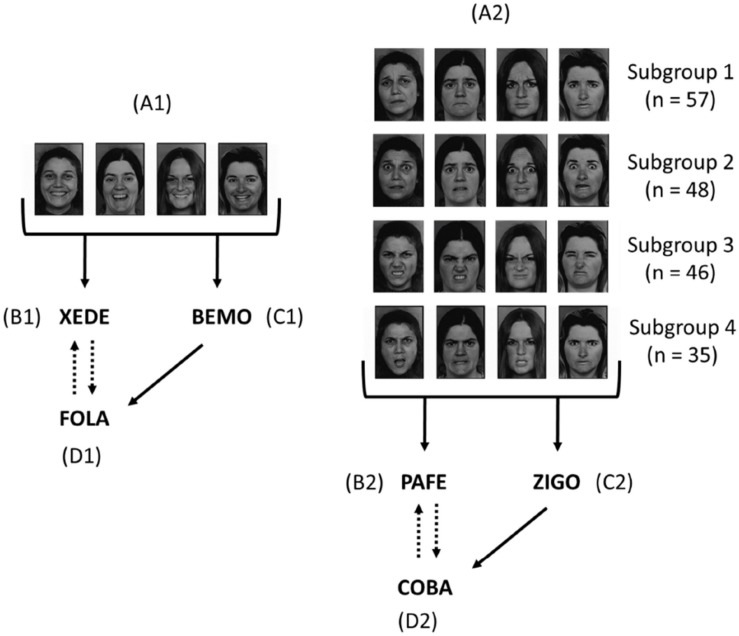
Diagram of the relational training employed in Phase 1. Solid arrows indicate relations directly taught; dashed arrows indicate derived relations tested in equivalence probes (see text for details). Stimulus set A is comprised of pictures of faces expressing emotions, and sets B, C, and D are comprised of meaningless pseudo-words. A1 is comprised of four happy faces and A2 of four non-happy faces, which expressed sadness for participants from subgroup 1, fear for participants from subgroup 2, disgust for participants from subgroup 3, and anger for participants from subgroup 4. Note that pseudo-words related to happy and non-happy faces were counterbalanced among participants.

Figure 1 presents the stimuli employed in the experiment. Set A was comprised of eight pictures: four happy faces (A1) and four non-happy faces (A2) that could express sadness, fear, disgust, or anger, depending on the participant’s subgroup. Sets B, C, and D were comprised of two nonsense words each. Each participant was submitted to a relational training to generate two equivalence classes, one comprised of pictures of happy faces and nonsense words and the other comprised of pictures of one type of non-happy faces and other nonsense words.

The pictures were extracted from the *Pictures of Facial Affect*^©^ CD-ROM, purchased from Paul Ekman’s website.^2^ Pictures of human faces depicting valid expressions of happiness, anger, disgust, fear, surprise, and sadness (Ekman and Friesen, 1976) are available in this CD-ROM.

### Procedure

#### Phase 1: Establishment of Equivalence Classes

Each matching-to-sample trial began with the presentation of the sample stimulus in the central window. A click on this window displayed two comparison stimuli, in two of the peripheral windows. The two other peripheral windows remained blank, and the sample remained in the central window. A click on the window containing the stimulus designated as correct produced a display of stars moving on the computer screen. Incorrect responses blackened the screen for 3 s. The consequence for a correct or an incorrect response ended the trial, and the next trial began after a 2-s inter-trial interval.

Participants learned the conditional discrimination AB first, with a block of 24 AB trials in which samples A1 and A2 were presented 12 times each in a randomized sequence. Sample A1 could be any one of the happy faces and sample A2 could be any one of the non-happy faces assigned for the participant (with expression of either sadness, fear, disgust, or anger). The positions of the comparison stimuli were determined according to a randomized sequence. In the first eight trials of this block a written prompt appeared on the screen. The Portuguese equivalent of the phrase “When this is here” appeared above the sample, and the equivalent of “Choose this” appeared above the correct comparison. These eight trials were followed by 16 trials without these prompts. If the learning criterion (correct choices in all 24 trials) was not achieved, the block was repeated. AB teaching ended when this criterion was attained, and then teaching of the AC relation began, with a similar procedure. When the participant made correct choices in all AC trials, CD training started, with a similar procedure. Each of these blocks – AB, AC, and CD – could be repeated for a maximum of three times. If the participant did not achieve the criterion in three presentations of a block, she or he was dismissed.

The next block verified maintenance of the cumulative baseline (AB, AC, and CD) and mixed 12 trials of each of these conditional relations, comprising, therefore, 36 trials in a randomized sequence. This block, with a different trial sequence, was repeated for a maximum of three times until the participant made no more than one incorrect selection.

When this criterion was achieved, the Portuguese equivalent of the message *The computer will no longer signal if your choices are correct or wrong* was displayed on the screen, and the cumulative baseline block (12 mixed trials of each conditional discrimination – AB, AC, and CD – totaling 36 trials) was repeated without differential consequences for correct and incorrect responses, until the participant made no more than one error. If the participant made incorrect choices in more than five trials, he or she returned to the cumulative baseline with differential consequences.

##### Equivalence probes

Two blocks of 16 probe trials without differential consequences tested equivalence-class formation. The first block evaluated the emergence of the BD derived relation. It was followed by the cumulative baseline block without differential consequences. Finally, the second probe block tested emergent conditional discrimination DB. These emergent conditional discriminations logically imply that trained conditional relations have the properties of symmetry and transitivity. Reflexivity is often assumed without tests in recent equivalence research. In addition, this arrangement permitted us to conduct the tests without the joint presentation of faces and words, which would be target and samples, respectively, in IRAP trials (see below). Figure 1 shows a schematic representation of the trained and tested relations in this phase.

In sum, participants were taught to establish two 4-member equivalence classes including both meaningful and arbitrary stimuli. The meaningful stimuli, designated as A1 and A2, were not individual stimuli; rather, each was comprised of four pictures of faces, with each face belonging to a different person. The common feature of the faces in each category was the emotional expression, which was a happy expression in A1 and a negative expression in A2 (see Figure 1). Different pictures were used to ensure that abstract stimuli would be equivalent to a particular emotional expression and not to idiosyncratic features of a particular face.

The next phase was designed to be performed primarily by participants who made no more than three errors in the two equivalence probe blocks. These participants met the criterion used to conclude that they formed the intended equivalence classes (i.e., one equivalence class containing the happy expression and three nonsense words, and another equivalence class containing one type of non-happy expression and three nonsense words). Some participants who did not achieve the equivalence criterion were also submitted to Phase 2, in order to compare IRAP data from participants that succeeded or not in equivalence-class formation.

#### Phase 2: The Implicit Relational Assessment Procedure (IRAP)

About 40% of the participants who demonstrated formation of equivalence classes were submitted to the IRAP immediately after the equivalence probes. The remaining 60% were submitted to the IRAP seven days after the equivalence probes. Participants who did not achieve the equivalence criterion were submitted to the IRAP seven days after the equivalence probes.

The IRAP trials were divided into “consistent” and “inconsistent” blocks. Appendix A presents the instructions for participants. On each IRAP trial, a sample, a target, and two response options were displayed on the computer screen. A sample word – either stimulus D1 (FOLA) or stimulus D2 (COBA) – was presented on the top of the screen; a single picture target – a happy or a non-happy face – appeared at the center; and the response options – V (for true) and F (for false) – were displayed at the two bottom corners of the screen (V on the left and F on the right^3^). All stimuli remained visible until the participant pressed one of the response keys. The task consisted of choosing one of these options by pressing either the “d” or the “k” key, corresponding to V (true) or F (false), respectively. The choice of the option considered correct removed all stimuli from the screen and, after 400 ms, the next trial was presented. The choice of the option considered incorrect produced a red X in the middle of the screen (immediately below the target picture). The next trial was presented only after the participant pressed the correct key.

**FIGURE 2:**
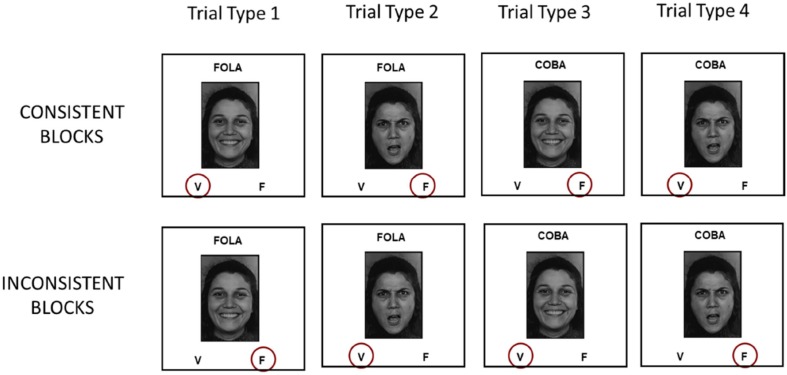
Schematic illustration of the four trial types presented during the IRAP blocks.

All participants were presented with blocks of 24 trials – at least two practice blocks and six test blocks. The practice blocks were repeated until at least 80% correct choices had been made consecutively in one consistent and one inconsistent block. Within each block, the target stimulus could be either a happy face or a non-happy face, in a randomized sequence, with the restriction that the target was happy in 50% of the trials and non-happy in the other 50%. The happy face could be one of the four pictures in this category, in a randomized order, so that each of the specific happy faces appeared four times. The same applied to the non-happy faces. In the consistent blocks, trials that presented FOLA as sample and a happy face as target, and trials that presented COBA as sample and a non-happy face as target, both demanded the choice of the option V, whereas F was the correct choice in trials that presented FOLA as sample and a non-happy face as target, and in trials that displayed COBA as sample and a happy face as target. Incorrect choices caused a red X to be presented below the face, and the participant had to make the correct selection in order to advance to the next trial. In the inconsistent blocks, the opposite responses were required. Figure 2 illustrates the four different trial types in this part of the experiment.

After completion of all practice blocks and the six test blocks, a written message indicated the end of the experiment. The participant was thanked, debriefed, and any questions about the experiment were answered.

### Data Analysis

The most important IRAP data is the “response latency”, defined as the time in milliseconds (ms) that elapses between the beginning of the trial and the correct response by the participant. In line with contemporary research involving measurement of implicit bias, IRAP latency data were transformed into “D scores”, which minimized the impact of factors such as age, motor skills, and/or cognitive ability of participants (Greenwald et al., 2003). In this study, the transformation was achieved by means of an adapted version of the D algorithm developed by Greenwald et al. (2003) for the Implicit Association Test. This adaptation of the D algorithm for the IRAP is called D-IRAP. The transformation of latency data into D-IRAP scores allowed us to infer differences between conditions aside from contamination, which comes from individual differences associated with extraneous factors.

All latency data were processed by the D-IRAP algorithm, available in the IRAP software. The algorithm processed the data as follows: (1) latencies obtained in training trials were discarded, and only latencies from tests blocks were used; (2) latencies above 10,000 ms were excluded from the analyses; (3) participants who presented more than 10% of test-block trials with latencies less than 300 ms were excluded from the study; (4) standard deviations for the four trial types were computed: four for the response latencies from Test Blocks 1 and 2, four from Test Blocks 3 and 4, and four more from Test Blocks 5 and 6 – a total of 12 standard deviations; (5) twenty-four mean latencies were calculated, one for each trial type in each test block; (6) difference scores were calculated for each of the four trial types, for each pair of test blocks, by subtracting the mean latency of the consistent block from the mean latency of the corresponding inconsistent block; (7) each difference score was divided by its corresponding standard deviation calculated in step 4, generating one D-IRAP score for each trial type for each pair of test blocks: 12 D-IRAP scores in total; (8) four trial-type D-IRAP scores were calculated by averaging the scores for each trial type across the three pairs of test blocks; (9) a final D-IRAP score (overall D-IRAP) was produced by averaging the 12 trial-type D-IRAP scores from step 7 (Timko et al., 2010).

Finally, latencies for the consistent blocks were subtracted from latencies for the inconsistent blocks. Thus, positive D-IRAP scores indicate that the participants responded faster in the consistent blocks; negative D-IRAP scores indicate that participants responded faster in the inconsistent blocks. A higher D-IRAP score indicates a larger difference in response latencies between consistent and inconsistent trials.

## Results

One-hundred-thirty-five participants (72.6% of total) showed formation of equivalence classes (Phase 1) and attained criteria for the IRAP (Phase 2). Fifty-two of them completed the IRAP right after the equivalence training and the other 83 completed the IRAP seven days after the equivalence training. Table 1 presents these numbers distributed by subgroup of participants.

**TABLE 1:** Number of participants in each subgroup who achieved both equivalence and IRAP criteria.

**Subgroup**	**Completed the IRAP**		**Total**
	**Right after**	**7 days later**	
(1) Happy and sad faces	17	28	45 (33.3%)
(2) Happy and fearful faces	11	19	30 (22.2%)
(3) Happy and disgusted faces	11	23	34 (25.2%)
(4) Happy and angry faces	13	13	26 (19.3%)
Total	52	83	135
	(38.5%)	(61.5%)	(100%)

Twenty-four participants who did not achieve the equivalence criteria (12.9%) completed the IRAP correctly seven days after the relational training. The remaining 27 participants (14.5%) did not produce IRAP scores (they either did not reach the IRAP criteria in the practice block or did not return to the experiment seven days later) and will not be considered in the following analysis.

### Overall D-IRAPs

The overall D-IRAP scores produced by participants who established the equivalence classes were positive and significantly different from zero [Overall D-IRAP_right after_: Mean = 0.13, *SD* = 0.19, *t*(51) = 4.94, *p* < 0.0001; Overall D-IRAP_7__days_: Mean = 0.11, *SD* = 0.22, *t*(82) = 4.41, *p* < 0.0001]. These results indicate that, in general, participants who established equivalence classes responded faster in the consistent than in the inconsistent IRAP conditions. On the other hand, participants who did not establish the experimental classes produced a mean overall D-IRAP that was not significantly different from zero [Overall D-IRAP_no equivalence_: Mean = 0.08, *SD* = 0.29; *t*(23) = 1.42, *p* = 0.17]. This result indicates that, in general, these participants took similar times to respond under the consistent and inconsistent IRAP conditions.

A one-way ANOVA showed a significant effect for the difference between the mean overall scores presented above [*F*(2,156) = 8.23, *p* = 0.0004]. To extend the interpretation of this result, a Tukey-Kramer multiple-comparison test was conducted and the results of this *post hoc* test are presented in Table 2.

**TABLE 2:** Results from the Tukey–Kramer Multiple Comparisons Test.

**Comparison**	**Mean difference**	***Q***	***p*-value**
No equivalence vs. Right after	−0.217	5.428	<0.001
No equivalence vs. 7 days	−0.196	5.228	<0.001
Right after vs. 7 days	0.021	0.723 ns	>0.05

*If the value of *q* is greater than 3.352, then the *P*-value is less than 0.05. ns = non-significant.*

A significant difference was observed between the overall mean D-IRAP scores produced by participants who established equivalence classes and by participants who did not establish these classes. There was no significant difference between the overall D-IRAP scores produced immediately after class establishment and the scores produced seven days later.

### Overall D-IRAPs From Different Subgroups

Out of the 135 participants with equivalence-consistent performances, 45 established classes involving happy and sad faces, 30 established classes involving happy and fearful faces, 34 established classes involving happy and disgusted faces, and 26 established classes involving happy and angry faces (see Table 1). The mean overall D-IRAPs from these subgroups were all positive and significantly different from zero, as shown in Table 3.

**TABLE 3:** One-sample *t*-tests calculated for the mean overall D-IRAPs generated by the participants of each subgroup.

**Subgroup**	**Overall D-IRAP**	***SD***	***t*- and *p-*values**
Happy and sad faces	0.110	0.21	*t*(44) = 3.53, *p* < 0.001
Happy and fearful faces	0.180	0.19	*t*(29) = 5.16, *p* < 0.0001
Happy and disgusted faces	0.081	0.23	*t*(33) = 2.04, *p* < 0.05
Happy and angry faces	0.117	0.23	*t*(25) = 2.56, *p* < 0.01

A one-way ANOVA showed that these mean general scores were not significantly different from each other [*F*(3,131) = 1.12, *p* = 0.34], indicating that the type of negative emotional expression had no significant differential impact on the magnitudes of D-scores. Therefore, the different non-happy faces will be henceforth referred to collectively as ”negative expressions”.

### D-IRAPs for Different Trial Types

**FIGURE 3:**
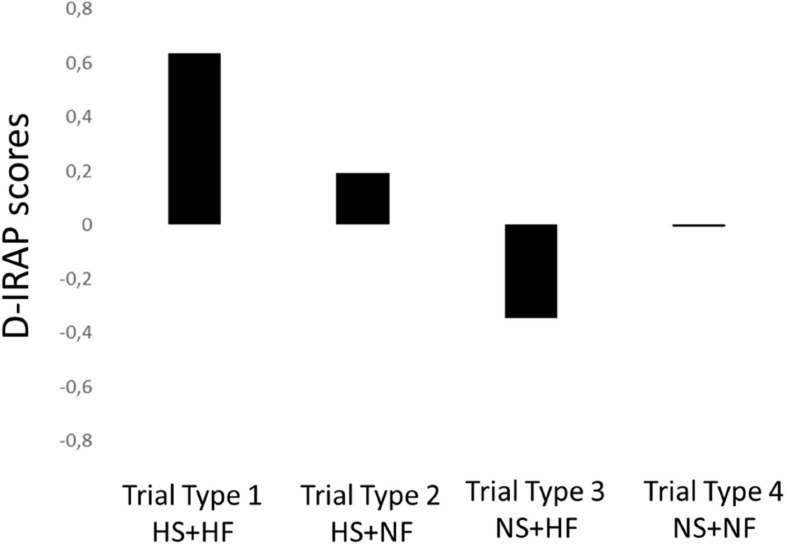
D-IRAP scores for each trial type extracted from the performances of the 135 participants who achieved both the equivalence and the IRAP criteria. HS stands for happy symbol, HF for happy face, NS for negative symbol, and NF for negative face.

The mean overall D-IRAP scores produced by the 135 participants appear to have been more significantly impacted by the participants’ performance in trials involving happy faces and their symbol (equivalent word) than by trials involving negative expressions and their symbol, as shown in Figure 3.

Participants were significantly faster to respond in the consistent blocks of trial types 1 and 2, slower in the consistent blocks of trial type 3, and did not show significant differences between the times to respond in the consistent and inconsistent blocks of the trial type 4. Table 4 presents the mean D-IRAPs scores for each trial type confronted with a value of zero.

**TABLE 4:** One-sample *t*-tests calculated for the mean D-IRAP scores from the four types of trials.

**Trial type**	**D-IRAP**	***SD***	***t-* and *p-*values**
1: happy symbol + happy face	0.64	0.42	*t* (134) = 17,44, *p* < 0.00001
2: happy symbol + negative face	0.19	0.37	*t* (134) = 6.04, *p* < 0.0001
3: negative symbol + happy face	−0.34	0.48	*t* (134) = 8.39, *p* < 0.0001
4: negative symbol + negative face	−0.005	0.39	*t* (134) = 0.16, *p* > 0.05

A repeated measures ANOVA showed a significant effect for the difference between the mean D-scores that participants produced for the IRAP trial types [*F*(3,134) = 132.75, *p* < 0.0001]. To extend the interpretation of this result, a Tukey-Kramer multiple-comparison test was conducted and the results of this *post hoc* test are presented in Table 5. Significant differences were observed between all the mean D-IRAP scores produced by participants who established equivalence classes.

**TABLE 5:** Results from the Tukey–Kramer Multiple Comparisons Test considering the four IRAP trial types.

**Comparison**	**Mean difference**	***Q***	***p*-value**
Type 1 vs. Type 2	0.442	12.427	<0.001
Type 1 vs. Type 3	0.982	27.593	<0.001
Type 1 vs. Type 4	0.641	18.002	<0.001
Type 2 vs. Type 3	0.540	15.167	<0.001
Type 2 vs. Type 4	0.198	5.575	<0.001
Type 3 vs. Type 4	-0.341	9.592	<0.001

*If the value of *q* is greater than 3.65, then the *P-*value is less than 0.05.*

### Time Elapse From Equivalence Tests and IRAP

**FIGURE 4:**
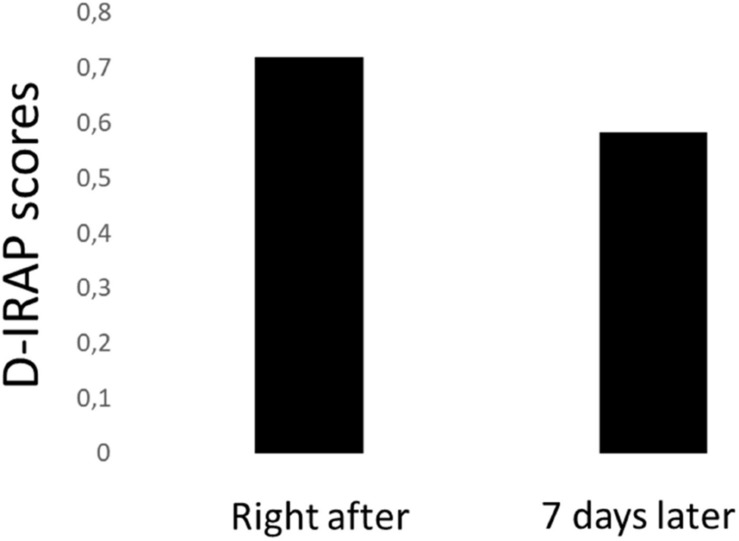
D-IRAP scores for trials involving the simultaneous presentation of the happy symbol and the happy face (trial type 1) produced right after and seven days after relational training and tests.

Time between equivalence tests and IRAP had a marginally significant effect on the modulation of D-IRAP for trial type 1 [Type 1_ right after_: Mean = 0.72, *SD* = 0.35; Type 1_ 7 days_: Mean = 0.58, *SD* = 0.46; *t*(133) = 1.83, *p* = 0.069], as depicted in Figure 4.

Time elapsing had no significant impact on the D-IRAPs extracted from trial type 2 [Type 2_ right after_: Mean = 0.22, *SD* = 0.41; Type 2_7__days_: Mean = 0.18, *SD* = 0.34; *t*(133) = 0.58, *p* = 0.56], trial type 3 [Type 3_right after_: Mean = −0.34, *SD* = 0.50; Type 3_7 days_: Mean = −0.35, *SD* = 0.47; *t*(133) = 0.09, *p* = 0.93], and trial type 4 [Type 4_right after_: Mean = −0.06, *SD* = 0.39; Type 4_ 7__days_: Mean = 0.03, *SD* = 0.38; *t*(133) = 1.43, *p* = 0.15].

Note: The data supporting the conclusions of this manuscript will be made available upon request to the first author.

## Discussion

The IRAP has typically been employed to measure the strength of derived relational responding in socially loaded contexts. The present study was different in the sense that it attempted to measure stimulus relations established in the laboratory after nonsense words were made equivalent to happy facial expressions and different types of negative expressions. When responding to the IRAP, differences in response latencies between *consistent* and *inconsistent* relations reveal behavioral biases frequently attributed to the learning history of the participants. The current study demonstrates that the IRAP effect is also sensitive to the nature of the stimuli presented through the trials and to experimentally induced, derived relations. Moreover, this study is consistent with the claim that the participant’s performance is multi-determined by the stimulus functions interacting with stimulus relations during the course of the IRAP trials (see Finn et al., 2018). In this section, we discuss how functions of certain stimuli may have even more impact on the direction and magnitude of the IRAP effect than the relational coherence previously learned by the participant.

The most important data presented in this article are the D-scores for the four different trial-types, which showed combinations among faces expressing happy or negative emotions and pseudo-words indirectly related to these faces. Only trial-types 1 and 2 yielded positive scores (i.e., faster responses in the consistent blocks, which required the participants to choose *true* when faced with the combination happy symbol–happy expression and *false* when faced with happy symbol–negative expression, respectively). D-IRAP scores for the trial-type 1 were significantly larger than the scores for the trial-type 2. For trial-type 3 (negative symbol–happy face = *false* in the consistent blocks), D-IRAP scores were negative, and scores were close (on average, statistically equal) to zero for trial-type 4 (negative symbol–negative face = *true*). The negative D-IRAP in trial-type 3 was particularly intriguing because it means that participants were faster to respond *true* than *false* for the combination of negative symbol and happy face; it suggests that the nature of the stimuli displayed in such a trial might have had more influence on the participants’ performance than the relational coherence derived from their previous experimental learning histories. In the following paragraphs, we attempt to explain these results and explore their implications.

Once the IRAP is a procedure that requires the participant to respond accurately and quickly, differences in recognition of any stimuli present in the experiment may contribute to stronger or weaker effects. In this sense, the happy faces used in the current study were probably easier to detect and to recognize than the negative faces, in both the equivalence training and the IRAP trials. This hypothesis is consistent with studies that report a *happiness superiority effect* in visual search paradigms (Becker S. et al., 2011; Craig et al., 2014; Lee and Kim, 2017) as well as in categorization processes (Leppänen and Hietanen, 2003). Using visual search paradigms, a broad range of studies suggests that happy expressions may be easier to detect than negative expressions (Juth et al., 2005; Calvo and Nummenmaa, 2008; Becker D. V. et al., 2011), at least when faces from the Pictures of Facial Affect database (Ekman and Friesen, 1976) are used (Tottenham et al., 2009). We have already reproduced this happiness superiority effect in our laboratory by measuring, in a visual search paradigm, the response latency and the number of ocular saccades needed to identify happy and angry targets as a function of the orienting function of these targets (Pinto, 2018). The happy face advantage in categorizing processes refers to the verification that happy faces are categorized faster as *happy* than, for example, angry faces are categorized as *angry* (Leppänen and Hietanen, 2003).^4^ Taking that into account, and consistent with the claim that emotionally salient stimuli can influence how attention is allocated (Fenske and Raymond, 2006), we assume that the orienting function of the happy faces might have played a critical role in the IRAP effects that can be inferred from the D-IRAP scores.

The role of the experimental stimuli that we chose to present as the response options in the IRAP trials also requires contemplation. It seems plausible to consider that the orienting function of the response option *true* may have been stronger than the orienting function of *false* (*true* may have a stronger orienting function since it frequently serves as a confirmatory, favorable, or positive response in natural language). Indeed, previous research (e.g., O’Shea et al., 2016) has shown that participants may find it easier to respond *true* to positive stimuli than to press *false*, if these are the IRAP response options. Based on the current results, it is possible to suggest that a behavioral bias to relate happy faces and *true* have emerged in the course of the IRAP trial types that presented such a combination (i.e., trial-types 1 and 3). This way, this conceivable behavioral bias may have affected the participants’ responses when happy and *true* were presented, by facilitating pressing the *true* key frequently faster.

Taking the above into account, we assume that relevant properties of the experimental stimuli, combined with the equivalence relations previously established, allow for an interpretation of the observed results. Once the pseudo-words FOLA and COBA were indirectly related to the faces through equivalence, probably some of the functions of the faces expressing emotions were transferred, and this effect was also critical for the results observed. When the *positive* pseudo-word was presented with a happy face (trial-type 1), the relational coherence between these equivalent stimuli may have been added to the hypothesized bias to relate the happy faces and *true*, resulting in a very robust positive D-IRAP score (i.e., faster responses in the consistent blocks). The bias to relate happy faces and *true* seems to have overcome the effect of the relational training on IRAP performances. In trial-type 3, when the *negative* pseudo-word was presented with the happy face, participants responded faster to the *true* relative to the *false* option, yielding thus a negative D-IRAP score (i.e., faster responses in the inconsistent relative to the consistent blocks). It could be that the negative pseudo-word had not acquired properties of the negative faces robustly during the relational training, and this may have contributed to diminishing the control of this stimulus. Notwithstanding this conjecture, it is possible that the mean negative score registered for the trial-type 3 would have been even more negative in the absence of the relational training. Additional research is needed to investigate such a possibility; in the absence of a baseline measure, it is not possible at this time to determine the precise impact that the relational training had on the IRAP performances. Further investigation on the role of the response options in studies such as the current one is also necessary. For instance, if we employed *similar* and *different* instead of *true* and *false*, would we get different results?

For the trial-types that presented the faces expressing negative emotions (trial-types 2 and 4), the effects were different. The positive D-IRAP score for trial-type 2, which presented the positive pseudo-word and the negative faces, indicates that participants performed faster in rejecting this relation (i.e., by responding *false* in the consistent blocks) than in confirming this relation (by responding *true* in the inconsistent blocks). If the positive pseudo-word acquired functions of the happy faces to some degree, we should expect a competition between the tendency to respond to *true* (based on the functions transferred from the happy expressions) and the tendency to respond to *false* (since the relation between label and target do not cohere with the experimental learning history). In such a case, coherence with the experimental history seems to have prevailed, since pressings of the *false* key were frequently faster. Considering the trial type 4, an analysis of performances indicates a probable indifference of the participants to the equivalence relation between the negative pseudo-word and the negative faces. The mean D-IRAP score produced for this trial type was statistically equal to zero, which means similar latencies to respond *true* for the relation between the negative pseudo-word and the negative expressions in consistent blocks and to respond *false* to the same relation in inconsistent blocks. This pattern suggests that the relational coherence between the negative pseudo-word and the negative faces was weak for the participants, even though the establishment of this derived relation during the equivalence probes was demonstrated. These results indicate that the relatively small number of trials in relational training may have established a relational coherence that was probably stronger in the happy class and weaker in the negative class, a finding that is consistent with previous work by our research group (e.g., Bortoloti and de Rose, 2009, 2011b, 2012; Bortoloti et al., 2013). Apparently, the relational strength increases with the number of trials in relational training, as evidenced by Bortoloti et al. (2013). Future research may systematically investigate how experimental parameters that favor the establishment of stronger equivalence relations would influence the D-IRAP scores.

On balance, the happy expressions seem to have influenced the IRAP scores more than the negative expressions. The influence was even stronger when the relation between the happy expression, presented as target, cohered with the happy pseudo-word, presented as label. Additional investigation should elucidate whether such an influence in this sort of experiment may be modulated by variables such as stimuli gender and ethnicity. Several studies have demonstrated that attributes such as sex, ethnicity, and age can influence the perception of the emotion displayed by the face and its orienting function (e.g., Hugenberg and Sczesny, 2006; Becker et al., 2007; Aguado et al., 2009; Hess et al., 2009; Lipp et al., 2015). As an illustration, when both female and male faces expressing anger and happiness are categorized, a happy-categorization advantage is often observed for female faces, whereas for male faces the happiness advantage is typically attenuated or even reversed (Hugenberg and Sczesny, 2006; Becker et al., 2007; Bijlstra et al., 2010). Furthermore, the critical role of stimulus selection for the observation of the happiness superiority orienting function in visual search has been often highlighted (Savage et al., 2016). Considering that the pictures selected for this study came from a single database (Ekman and Friesen, 1976) and only white female posers were presented to the participants, further investigation is necessary to assess the impact of different facial attributes on the symbolic relational responding.

### Stability of Equivalence Classes Including Happy Faces

In this study, the time elapsing between the equivalence tests and application of the IRAP marginally decreased the mean score for the IRAP trial-type 1, involving the happy symbol and the happy expression, but this trial type still dominated over the other trial types. This finding seems consistent with those reported by Silveira et al. (2016), in a study that investigated the stability of equivalence relations and transfer of functions over time. The authors showed that an equivalence class that included happy faces and arbitrary forms proved to be more stable than an equivalence class that included angry faces and arbitrary forms. Specifically, the number of participants who maintained the happy class was nearly double the number of those who maintained the angry class in a test conducted 30 days after the original relational training. The happiness superiority effect reported by Silveira et al. (2016) is consistent with the cognitive literature that claims a special place for happy faces in long-term memory processes (e.g., D’Argembeau and Van der Linden, 2007, 2011). In a typical old/new paradigm, happy faces are often more accurately identified, compared to angry, fearful, neutral, surprised, and disgusted faces. Some authors even predicted that the more positive the valence of an expression shown by a face, the better the recognition of this face in long-term memory tasks (D’Argembeau et al., 2003). This means that the best performance is predicted for happy expressions, followed by neutral ones, which may in turn be superior to negative expressions. The results reported here and by Silveira et al. (2016) are consistent with this predictive hypothesis.

## Conclusion

Participants of the current study produced positive overall D-IRAP scores, but considering the variability of outcomes observed for each trial type (one very strong and positive, one strong and negative, one mild and positive, and one close to zero), any conclusion based solely on these overall scores would not be reliable. In this sense, we believe that the interpretation of the individual trial types provided a more accurate explanation for the behavior observed during the experiment. Broadly speaking, the D-IRAP scores were more influenced by the happy expressions than by the negative expressions. This difference is consistent with a *happiness superiority effect*, as described earlier. Further experiments can present more evidence of the critical role of stimulus selection for both equivalence and IRAP studies.

## Ethics Statement

All procedures performed in this study were in accordance with the ethical standards of the Brazilian National Health Council. The protocol was approved by the Federal University of Minas Gerais ethical committee. Participants were informed through a disclosure statement, provided to them at the beginning of the study, that they would serve as participants in an experimental simulation of symbolic relations. Participants were informed that their completion of the study was part of their training as experimental researchers since they would use similar procedures along the semester in their own experiments, but they could quit their participation at any time. Information on all known risks and benefits of the study as well as confidentiality procedures was provided.

## Author Contributions

RB and JdR contributed conception and design of the study. RB and RdA collected data and organized the database. RB performed the statistical analysis and wrote the first draft of the manuscript. JdA contributed to discussion of the results. All authors contributed to manuscript revision, read, and approved the submitted version.

## Conflict of Interest Statement

The authors declare that the research was conducted in the absence of any commercial or financial relationships that could be construed as a potential conflict of interest.

## Footnotes

1 https://osf.io/kg2q8/

2 www.paulekman.com

3 The most common Portuguese words for true and false are “verdadeiro” and “falso”, with initials V and F, respectively.

4 Early experiments of the happy-face advantage described it as a general phenomenon. More recently, however, differences in the size of the happy-face advantage across pictures differing in ethnicity (Hugenberg, 2005) or gender (Hugenberg and Sczesny, 2006) have been investigated (Refer to discussion below).
